# Ophthalmia

**Published:** 1839-01

**Authors:** 


### ART. XIII.

*Ophthalmia. The various Inflammations of the Conjunctiva or Mucous Membrane of the Eye.* By J. SLADE, M.D.—*London,* 1838. 8vo. pp. 120.

WE are of opinion that Dr. Slade has committed an uncalled-for and useless act of injustice to his own work, by inserting the following paragraph in the title-page: "This treatise will be found useful to country families, and particularly to those persons who prescribe for the sick poor in the absence of medical assistance." This was probably an afterthought, or the suggestion of some well-meaning friend; for we observe the title-page has been printed separately from the body of the work,—in fact, reprinted. Dr. S.'s essay is not at all of a popular cast, and would prove supremely useless in the hands of any one but a medical practitioner: it is full of technicalities, and utterly beyond the conception of any one unacquainted with anatomy and pathology. What would Lady Bountiful make of the projected experiment of inoculating the eyes of twenty infants with gonorrhœal matter? (p. 72;) or what would be her feelings on reading that "there are few systems unmixed with scrofula, scurvy, or syphilis?" (p. 14.)

Considered as an essay on the inflammations of the conjunctiva, the work is above mediocrity: it is a good repetition, in fact, in somewhat altered language, of what is to be found in all the modern English treatises on the diseases of the eye, without containing one single new pathological or practical idea. We cannot agree with the author that "the different inflammations of the conjunctiva have never been considered upon the plan pursued in the present treatise." Upon what other plan, we would ask, have they been considered by Lawrence, Mackenzie, and the other English systematists of the present day? These authors have considered them exactly on the plan followed by Dr. Slade.

Dr. S.'s style is often very faulty; for example, the following sentences, among many others: "When once this tunic [the cornea] is wholly opaque, so as to prevent the admission of light, there is no remedy for its removal." (p. 15.) "The curability of the disease depends much," "according to the violence of the degree, &c." (p. 16.) "Persons employed in iron-foundries, glass manufactories, in any occupation where heat is accompanied by reflected light, which is always worse than direct light, such as might proceed from the sun or lightning, are frequent promoters of conjunctivitis." (p. 28.) Such phrases as "sensitivity," (p. 4;) "increased action in the circulating medium," by circulating medium being meant the blood-vessels, (p. 8;) "desight,"

for deformity, (p. 41,) &c. are also objectionable: but let us turn to matters of more importance.

Dr. S. states (p. 2,) that "the conjunctiva is perforated by ducts, called ciliary ducts." Now, it is well known, and evident, indeed, on the slightest examination, that the Meibomian or ciliary ducts do not open in the conjunctiva, but in the skin, near the inner margin of the edge of the eyelid, (*margo internus limbi palpebræ.*) Speaking of the external lamina of the cornea which he calls *conjunctiva corneæ*, (p. 4,) Dr. S. tells us that its connexion with the cornea "is so firm, that maceration, dissection some time after death, and disease only, are capable of apprizing us that the membrane is extended over the cornea." He does not appear to be aware of the simple experiment of throwing the eye and eyelids into boiling water, which in a moment coagulates the external lamina of the cornea, while it has no such effect on the conjunctiva, raises it in blisters, and at once demonstrates the existence of a membrane continuous with the conjunctiva and investing the cornea, and the physical differences in the character of this membrane and the conjunctiva. Because one membrane is continued into another, and that even abruptly, we must not conclude that they are of the same nature. On the fringes of the *morsus diaboli* we have a mucous membrane abruptly joining a serous membrane. On the surface of the eyeball we have a membrane, not coagulable by heat, suddenly terminating in one which immediately coagulates, like white of egg, on being submitted to the action of boiling water. The latter is continuous with the conjunctiva, but is not conjunctiva. Its proper appellation is *epikeratoeides*. Haller (*First Lines of Physiology*, § D,) believed this membrane to be double; and probably he was correct.

While discussing (p. 8) the state of the vessels in conjunctivitis, Dr. S. remarks, that "here we find the tubes gorged or overloaded with those red particles of blood which they are not destined to carry in health." It is impossible to know whether he means that the vessels are gorged with an extra quantity of red blood, or that they in health carry only colourless blood, but are now filled with red globules. If the latter is his opinion, we believe it to be erroneous. Every physiologist knows that, in the fine capillary vessels, such as most of those of the sclerotic conjunctiva, those of the cornea and its tunic, and those of the crystalline capsule, the red particles flow one after another in a single series; that, when they flow thus singly, as each measures only the three-thousandth or four-thousandth part of an inch in diameter, the vessels in which they are moving are invisible to the naked eye; and that it is only when the red globules are accumulated, and are running numerously side by side, that the vessels appear red. Capillaries carrying colourless blood appear to have no existence in any part of the body.

Dr. S. (p. 34) speaks of hypopium occurring in corneitis, (certainly a very rare event,) and tells us that the matter is "supplied by the cornea through an ulcerous aperture of the posterior layer." Certainly we have seen this happen once or twice in scrofulous abscess of the cornea, but never in corneitis. The tendency of matter, deposited even in the depth of the cornea, is generally outwards; conformably to the common law, that abscesses extend towards the surface, and shun the cavities of the body. We suspect that abscess of the cornea is often mistaken for hypopium. In the inflammations of the conjunctiva, Dr. S. appears (p. 44) inclined to trust a good deal to the efforts of nature, which "is both willing (he says,) and able to do much towards repairing any mischief that disease may incur." We believe, on the contrary, that there is no class of diseases in which there is less tendency to a natural cure than the inflammations of the conjunctiva. Catarrhal ophthalmia, for instance, if neglected, readily passes into the granular stage; the consequence of which is, too often, nebulo-vascular cornea and permanently impaired vision.

The instrument best adapted for removing foreign particles adhering to the corneal conjunctiva or imbedded in that membrane, Dr. S. considers to be a cataract-needle. A small, elastic, silver spatula is less formidable to the patient, and detaches the foreign body more readily, with less pain, and less abrasion of the corneal covering. Dr. S. dissuades from venesection, unless the system be plethoric; and, especially if the conjunctivitis is scrofulous: he says (p. 51,) "it should be avoided, even if the inflammation is very active." On this point we differ entirely with our author. In active scrofulous inflammation of the conjunctiva, especially in adolescents or adults, venesection is both useful and necessary. Dr. S. recommends (p. 52) leeches to be applied chiefly to the external surface of the lids. The side of the nose, over the nasal vein, is a preferable situation: two or three leeches placed there generally cause as much discharge of blood as twice that number on the lids. In acute conjunctivitis, Dr. S. thinks we should seldom venture to scarify, (p. 52.) "unless it is by making one deep incision the length of the palpebra." The disadvantage of making a deep incision is, that the central ducts of the Meibomian glands are cut across, and probably become obliterated. We are friendly to scarification in all the mucous ophthalmiæ; but we try to limit the depth of our incision to the swoln conjunctiva, and avoid dividing the fine lamina of tarsus, which intervenes between the conjunctiva and the Meibomian glands. We have no fears respecting the effects of scarifying in gonorrhœal ophthalmia: Dr. S. has terrified himself unnecessarily (p. 53) about the specific virus coming in contact with the divided surfaces. Even the removal of a fold of the chemosed membrane is a good practice in that disease.

We must confess we have been somewhat startled at Dr. S.'s recommendation of Goulard water, Goulard extract, and other preparations of lead, in the ophthalmiæ, (see pp. 54, 67, 95.) To use the words of our author, "It need not be said they are to be abandoned." If any one doubts of this, let him look at the figures accompanying Dr. Jacob's paper in the fifth volume of the Dublin Hospital Reports. If there is the least excoriation or ulceration of the conjunctiva or of the epikeratoeides, there the lead is immediately precipitated and fixed, forming an opacity, which is in general indelible. Even the druggist's apprentices begin to know this, and refuse to sell sugar of lead to make a wash for a sore eye. In treating of the ophthalmia neonatorum, Dr. S. repeats his irrelevant objection to scarifying the conjunctiva, and recommends blisters (p. 84) only as a last resource. We have found a blister behind the ear of great service in this ophthalmia, even within two or three days of its commencement.

"Syphilis, whereof the nature of gonorrhœa partakes," (p. 90,) is a clause showing at once Dr. S.'s notions on these two diseases, and his bad style of writing; but which we cannot stop to criticise now.

Speaking of gonorrhœal ophthalmia, Dr. S. asserts, that "a singular circumstance connected with this disease is, that females are never its victims." Now, cases are recorded by Wardrop, Delpech, and Bacot, of women being inoculated with gonorrhœa in the eyes, from the use of infected sponges and the like;* so that this remark of Dr. S. must be limited to there being no recorded case of a woman inoculating her eyes from her own body directly; but there seems no impossibility of such an event. As to the treatment of gonorrhœal ophthalmia, Dr. S. tells us that some trust to a free antiphlogistic course, but that his experience decides in favour of the astringent plan. Undoubtedly, the true mode is to combine the two; to bleed generally and locally, and to wash the conjunctiva with a solution of from two to eight grains of lunar caustic in an ounce of distilled (not rose) water.

With regard to variolous ophthalmia, we would ask Dr. S. if he ever saw a variolous pustule on the cornea? We never did, and believe such a thing never happens. Dr. S. takes no notice of the secondary variolous ophthalmia, which so frequently destroys vision, by causing abscess and bursting of the cornea.

The case of Mary Flint (p. 113) appears to be neither more nor less than one of fungus hæmatodes; and we would recommend Dr. S. to withdraw the absurd statement, from his next edition, of the surgeon's having destroyed the sight of one eye while scarifying the lids of the other.

We cannot agree with Dr. S.'s remark, that in scrofulous ophthalmia "leeches are seldom demanded." (p. 117.) Nor can we yield assent to the assertion that "lotions have no specific virtues, if we except an evaporating one." (p. 118.) Leeches to the side of the nose and eyelids generally relieve in scrofulous ophthalmia; while a lotion of from one to two drachms of vinum belladonnæ in eight ounces of water, used warm, removes the intolerance of light in so remarkable a manner as to merit, we think, the appellation of a specific remedy.

We trust Dr. S. will take these criticisms in good part: they refer to small fractions of his book only. On the whole, we judge very favorably of his ophthalmological attainments, from a careful perusal of his essay. The diagnosis is pursued with minuteness and accuracy, and the practice recommended is, in general, highly judicious. We would particularly recommend to some of our modern systematists, Dr. S.'s distinction of simple mucous conjunctivitis from catarrhal ophthalmia, which are too much confounded; and to practitioners generally we would say, Read and study Dr. S.'s book.

* See Lawrence on Venereal Diseases of the Eye, p. 32.

